# Supplementary figures and images for: miR-155 exerts posttranscriptional control of autoimmune regulator (Aire) and tissue-restricted antigen genes in medullary thymic epithelial cells
Source: BMC Genomics. 2022 May 28;23:404. doi: 10.1186/s12864-022-08631-4 (PMC9145475; doi:10.1186/s12864-022-08631-4)

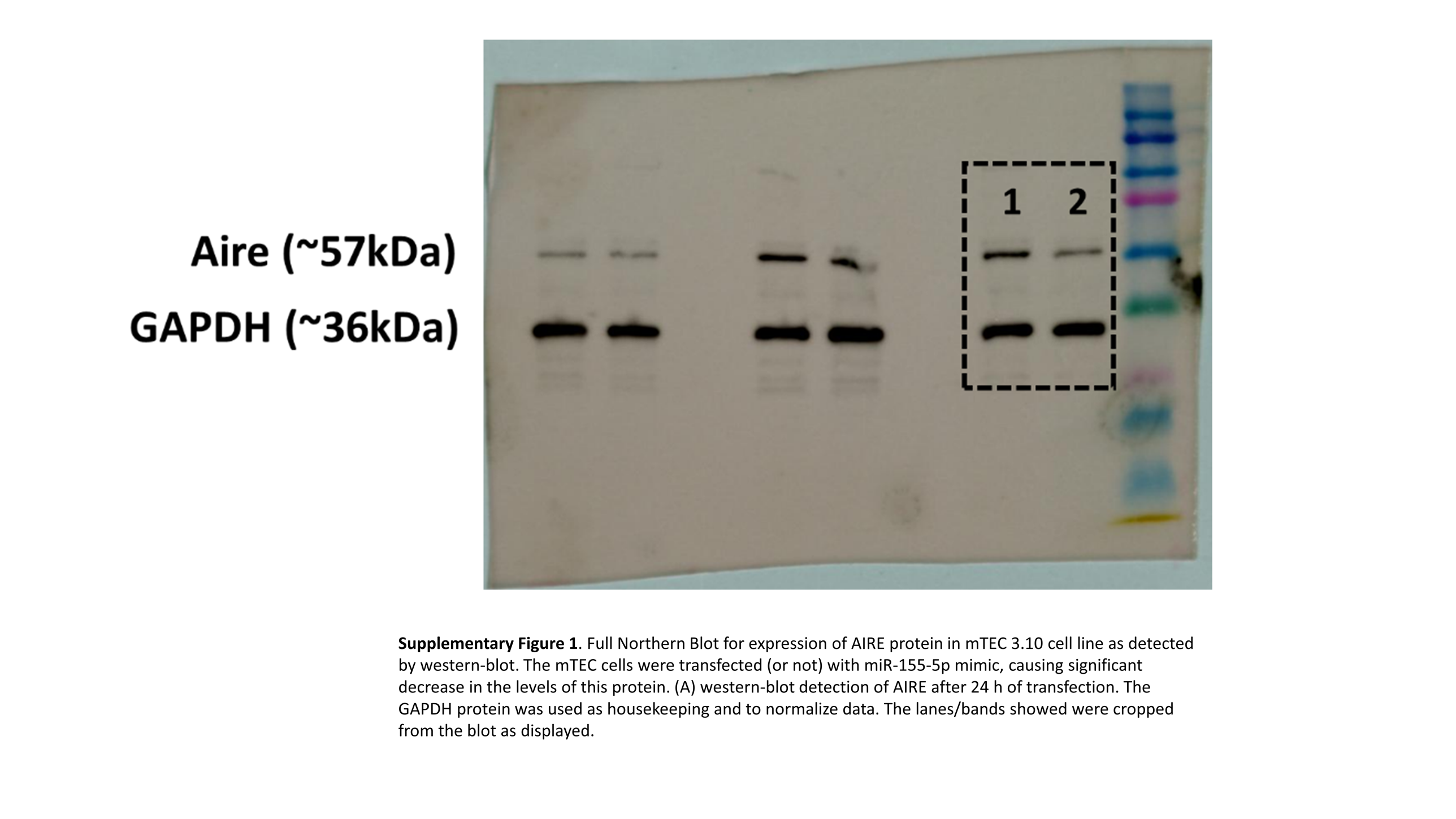

Supplement: Supplementary file 1 — Additional file 1. [file 12864_2022_8631_MOESM1_ESM.tif]

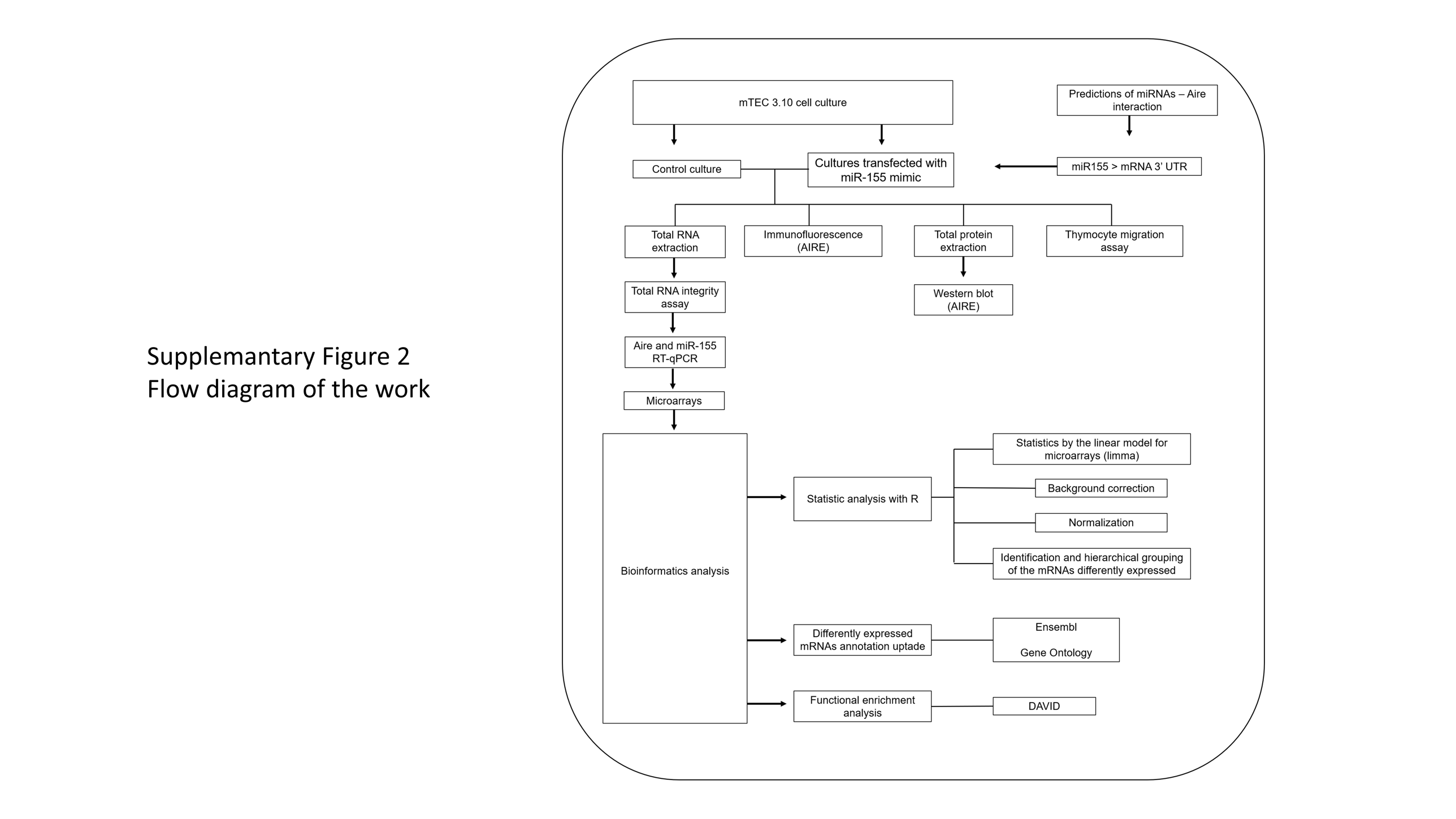

Supplement: Supplementary file 2 — Additional file 2. [file 12864_2022_8631_MOESM2_ESM.tif]
